# Monthly Summary

**Published:** 1897-10

**Authors:** 


### Monthly Summary.

ACETANILIDE AS A DRESSING.—Morton writes in the *Polyclinic* of February 16, 1895, that the action of acetanilide upon wounds, especially granulations, when used in full strength, is to produce intense dryness, blueness, and to check at once and to prevent the formation of pus. Upon extensive granulating surfaces and chronic ulcers a slight burning sensation is at first perceived, which is rapidly succeeded by a sedative or anesthetic effect. If used in sufficient quantity, a thin scab of acetanilide, combined with the wound secretions, forms under which healing rapidly progresses. If a very large surface is exposed to the action of the undiluted drug, toxic symptoms promptly supervene in susceptible individuals. It is probable that children and the aged are more sensitive to its absorption than are vigorous middle-aged persons. It is also probable that anæmia might follow too prolonged application of large quantities of the substance, because of its destructive action upon the red blood-corpuscles; this, however, he has not seen. The powder does not, as a rule, stick to wounds or hold dressings fast; but when it does so, alcohol causes instant release by dissolving the drug.

Under no circumstances does acetanilide irritate the skin or wounds, even when used beneath impervious protectives or antiseptic poultices.

What may be the best vehicles for applying acetanilide must yet be proved. Upon most of his cases the pure powder was used from a dusting-box. This, while usually safe, he thinks, has been an unnecessary waste, for very recent experiments in dilution have shown him that a one-fifth to one per cent. mixture with petrolatum was sufficient to arrest suppuration and secure rapid healing in an extensive septic scald. All pain vanished after the first application.

Acetanilide dissolves in five volumes of alcohol, in twenty volumes of ether, and in two hundred volumes of water. It is soluble in liquid petrolatum to the extent of forty grains to the ounce. In chloroform it very freely dissolves.

By diluting with water a saturated alcoholic solution of acetanilide, the drug will be thrown out of solution in the shape of fine crystals, and will remain perfectly mixed in suspension long enough to permit of its use in this form as an injection for abscesses or carbuncles, in gonorrhea, etc.

In the large number of cases in which he has freely employed acetanilide, but twice have toxic effects been noticed; one was in an infant aged fourteen months.—*The Therapeutic Gazette.*

---

A SIMPLE WAY OF PUTTING ON METAL BACKINGS FOR VULCANITE WORK WITHOUT SOLDERING.—By Dr. Kessell.—We make use of this plan in partial cases where patients are liable to break off the porcelain, leaving the pins in the plate. Any metal of gauge 27 or 28, either gold, platinum or Columbian silver (he uses the latter exclusively) may be used. After the plate tooth is ground in position, a piece of the metal, the width of the tooth and one-half to three-quarters of an inch in length, is first annealed, then holes cut in it to accommodate the pins of the tooth. The metal is slipped over the pins, and they are bent to form an X. The tooth, with the metal in place, is then put back on the model, which is waxed up as usual. When the case is packed the rubber will surround the bent pins and the metal. The advantage of the backing is that it gives strength to the tooth, and it also allows the lower tooth to strike only against the metal instead of the thin porcelain. Rubber will vulcanize very well with Columbian silver, and it will hardly tarnish in the mouth. This process requires but a few minutes' work, and it removes the danger of checking the tooth or discoloring it by the heat required for soldering, nor is it necessary to have the case invested.—*Cosmos.*

---

A GOOD LAW.—In Bulgaria the proprietors of a medicine by which they claim to cure a specified disease are liable to be imprisoned if the medicine fails to produce the desired effect.—*Ohio Dental Journal.*

DISCUSSION ON LOCAL ANESTHESIA.—Dr. Haupt recommends the use of tablets of cocain prepared by Parker & Davis. The bottle in which the tablets are sold is a convenient measure for making the solution, one tablet in the bottle half full of water making a two per cent solution. The use of these tablets gives a solution always fresh and safe.

Dr. O. N. Heise has lately used eucain in ten per cent. solution, injecting about five drops as a local anesthetic. This seems absolutely safe, even with twice this amount. The effect is also more lasting than that of cocain.

Dr Otto Arnold uses eucain, and prefers it to cocain, as it appears to be free from toxic effects; but where teeth firmly imbedded are to be extracted there is nothing better than nitrous oxid. Statistics show there has been more fatalities from cocain than there had been from nitrous oxid, though the latter has been in use so much more generally and so many years.

Dr. L. L. Barber has better success with eucain than with cocain, and never has any alarming symptoms from its use. He thinks the amount of cocain that enters the system with the cataphoric administration was so small that no evil could follow its use. He has not used very strong solutions himself, but has heard of others using as high as seventy-five per cent. with no bad effect.

Dr. Snyder: It is probable that bad results arising from the use of the hypodermic syringe often arise from lack of care of the hypodermic needle. If there is carelessness, the result will be that microbes will be conveyed into the wound made and cause trouble. The needle should be thoroughly disinfected always before use, and care should be taken to avoid the periosteum. The surface of the gums also should be clean before the injection is made.

Dr. Jones: When it is necessary to extract teeth, we should use some means to avoid pain. It is a duty we owe to the human family. In making an injection he did not make it close to the teeth, but in the deep tissues, where it is easy to get. Cocain acts on the ends of the nerves of sensation.—*Cosmos.*

KAISERLING'S METHOD FOR THE PRESERVATION OF SPECIMENS WITH THEIR NATURAL COLORS.—The above method was referred to by Dr. W. F. Whitney, at the March meeting of the Boston Society of Medical Sciences.

Slices of organs from 3 to 5 ctm. thick are placed from 3 to 5 days in ;

| | | |
|---|---|---|
| 1. | Formaldehyd, - - | 200 c.c. |
| | Water, - - - | 1,000 c,c. |
| | Nitrate of potash, - - | 15 gme. |
| | Acetate of potash, - - | 30 gms. |

They are then removed, the fluid allowed to drain off and placed in :

2. Alcohol 80 per cent. for 6 hours, then
   Alcohol 95 per cent. for 2 hours.

From this directly into

| | | |
|---|---|---|
| 2. | Water, - - - | 2,000. |
| | Acetate of potash, - - | 200. |
| | Glycerin, - - - | 400, |

For permanent preservation in a dark place.

Further details for the preservation of whole organs, etc., should be studied in the original article (C. Kaiserling, Virch. Arch. Bd. 147s, 389).

The results thus obtained are certainly wonderful, and promise to be one of the greatest aids in teaching of this generation.—*American Medico Surgical Bulletin.*

---

MEDICAL SUPERSTITIONS.—For toothache trim your finger nails on Friday; eat bread a mouse has nibbed, or bury a tooth in the hole of a mouse, or carry in the pocket a tooth from a soldier killed in battle, or from a murdered man. Kiss a mule. Rub the gums with the body of an ant, bee or fly, or prick them with a sharp twig from a sweet apple tree.

---

A NEW HEMOSTATIC.—Ferripyrin—a combination of one part ferric chlorid, three parts anti-pyrin and five parts water, has great hemostatic properties. It has an agreeable taste, and is readily applied. One application usually suffices.—"Dental Digest."

TO OPEN PULP CHAMBERS OF TEETH AFFECTED WITH PERICEMENTITIS.—By R. E. Sparks, M. D., D. D. S., Kingston, Ont.—We often have to open up teeth which are very tender to the touch. The pressure necessary to make a steel drill enter the outer layer of enamel, together with the shocks caused by the revolutions of the flat sided drill upon the uneven surface of the tooth, causes excruciating pain. To avoid this, grind, with a small stone, a pit at the point at which you wish to enter the tooth. The drill will then run smoothly and penetrate much more easily. When desiring to open on the palatine surface of the incisors or canines, after grinding the pit, take an inverted cone bur, a little larger than the drill intended to be used, and cut into the pit the depth of its diameter. This gives a flat surface for the point of the drill to start into, and avoids the shocks before spoken of. Keep the point of the drill well lubricated with oil of turpentine or glycerine.—*Dominion Dental Journal.*

---

A HINT ON EXTRACTING.—B. E. A. Randall, D. D. S., Truro, N. S.—I have often seen an operator's face and shirt front bespattered with blood, from extracting teeth, while the patient was under an anæsthetic. This is unnecessary. I use nitrous oxide as a general anæsthetic. The moment that the inhaler is taken from the face I push a small sponge into the mouth; this prevents roots, which I may drop in the mouth, from being drawn back into the trachea, and absorbs the blood which would otherwise be swallowed or blown out in the operator's face, and does not prevent breathing, as the patient breathes through the nose.—*Dominion Dental Journal.*

---

AMMONOL.—Dr. Watkins, at the First District Dental Society, State of New York, reported in December *Cosmos*, described ammonol, one of the coal tar products, as the most satisfactory preparation he had used for pericementitis. In cases where a tooth has been filled for some time and becomes sore and elongated, ammonol given in 5 to 15 or 20 gr. doses will entirely remove soreness in a short time.

